# Photosynthetic capacity of senescent leaves for a subtropical broadleaf deciduous tree species *Liquidambar formosana* Hance

**DOI:** 10.1038/s41598-017-06629-7

**Published:** 2017-07-24

**Authors:** Zidong Luo, Huade Guan, Xinping Zhang, Na Liu

**Affiliations:** 1 College of Resource and Environment Science, Hunan Normal University, Changsha, 410081 China; 2 School of the Environment & National Centre for Groundwater Research and Training, Flinders University, Adelaide, SA 5001 Australia

## Abstract

Photosynthetic capacity and leaf life span generally determine how much carbon a plant assimilates during the growing season. Leaves of deciduous tree species start senescence in late season, but whether the senescent leaves still retain capacity of carbon assimilation remains a question. In this study, we investigated leaf phenology and photosynthesis of a subtropical broadleaf deciduous tree species *Liquidambar formosana* Hance in the central southern continental China. The results show that *L*. *formosana* has extended leaf senescence (more than 2 months) with a substantial number of red leaves persisting on the tree. Leaf photosynthetic capacity decreases over season, but the senescent red leaves still maintain relatively high photosynthetic capacity at 42%, 66% and 66% of the mature leaves for net photosynthesis rate, apparent quantum yield, and quantum yield at the light compensation point, respectively. These results indicate that *L*. *formosana* may still contribute to carbon sink during leaf senescence.

## Introduction

Forests form the most important carbon pool of the terrestrial ecosystems, thus play a significant role in global carbon balance. The recent decade has seen an increasing research interest on carbon assimilation and balance in tropical and temperate forests^1–5^. However, studies on ecological process (e.g. carbon uptake, water use) on subtropical forests are still limited until recently^6–11^. In the East Asian monsoon region, it distributes a typical subtropical forest ecosystem, which is composed of evergreen broadleaf, deciduous broadleaf and mixed stands^8^. Photosynthesis of forest trees in this region might be limited by temperature in winter, and by solar radiation in summer when other resources are abundant^9^. Nevertheless, the subtropical evergreen forests maintain high rates of photosynthetic activity (around 50% of peak values) in cold winter^7^. Considerably high winter carbon assimilation (5.4 to 8.8 μmol CO_2_ m^−2^ s^−1^) has been documented for ten subtropical evergreen broadleaf tree species^12^. Extended leaf senescence of deciduous species in this climatic zone can promote carbon assimilation in winter^9^. However, the subtropical deciduous tree species have attracted little attention regarding their photosynthetic capacity and potential carbon assimilation during leaf senescence.

Leaf senescence is a primary characteristic of deciduous species, with visual change in leaf pigmentation in autumn and winter. Trees need to store resources to fuel bud flush and shoot growth in the following spring^13^. Nutrients (e.g., carbohydrates) stored in the leaf require a continual supply of photosynthesis to support resorption^14^. Therefore, photosynthesis during leaf senescence is critical to plant physiological processes^14, 15^. Timing of leaf senescence can have a significant impact on ecosystem productivity^16^. An extended growing season provides extra time for plant photosynthetic activity, increasing carbon assimilation potential. Goulden *et al*.^17^ has documented that a delay in senescence for 5–10 days in a temperate deciduous forest can result in an increase of about 500 kg C ha^−1^ in the annual gross production. For subtropical deciduous broadleaf forest in China, a previous study shows that its growing season has prolonged from both an earlier onset of green-up and a delay in dormancy^18^. A good understanding of photosynthetic capacity of senescent leaves is essential to estimate the effects of the prolonged growing season on carbon assimilation for such deciduous species.

Temperature is regarded as an important driving factor on plant phenology. The length of growing season increases due to global warming^19–23^. Delays in vegetation dormancy onset date have been documented recently for a wide range of ecosystems in China^24^. Different deciduous tree species differ in leaf life span and duration of leaf senescence, which may lead to different impacts on late season (autumn and winter) carbon assimilation during leaf senescence. Zhang *et al*.^9^ show that red leaves of a subtropical deciduous tree species during leaf senescence maintain a relatively high photosynthetic rate (about 36% of peak values in summer) and contribute to carbon uptake in the late season. Whether or not such a late season photosynthesis pattern of senescent leaves is common for other deciduous species remains to be investigated.

Lack of observation-based studies on the deciduous species is responsible for the current poor understanding of photosynthesis of senescent leaves. This situation also limits our capacity to model the carbon fluxes of forest ecosystems. It is reported that most of the published models for the terrestrial carbon cycle fail to capture the response of tropical carbon fluxes to climate variability^25^. Such problems may be associated with biases in the estimated response of productivity or ecosystem respiration to climate^5^, or the misrepresentation of photosynthetic process among different plant functional types (e.g., evergreen and deciduous species) in the models.

In this study, we investigate leaf photosynthesis and phenology of a common subtropical deciduous tree species, *Liquidambar formosana* Hance, in the field in Hunan Province, the central southern China. The primary objectives are to investigate temporal variation of photosynthetic capacity of *L*. *formosana* and to examine whether senescent leaves still retain capacity for carbon assimilation.

## Results

### Leaf development and senescence

**Figure 1:**
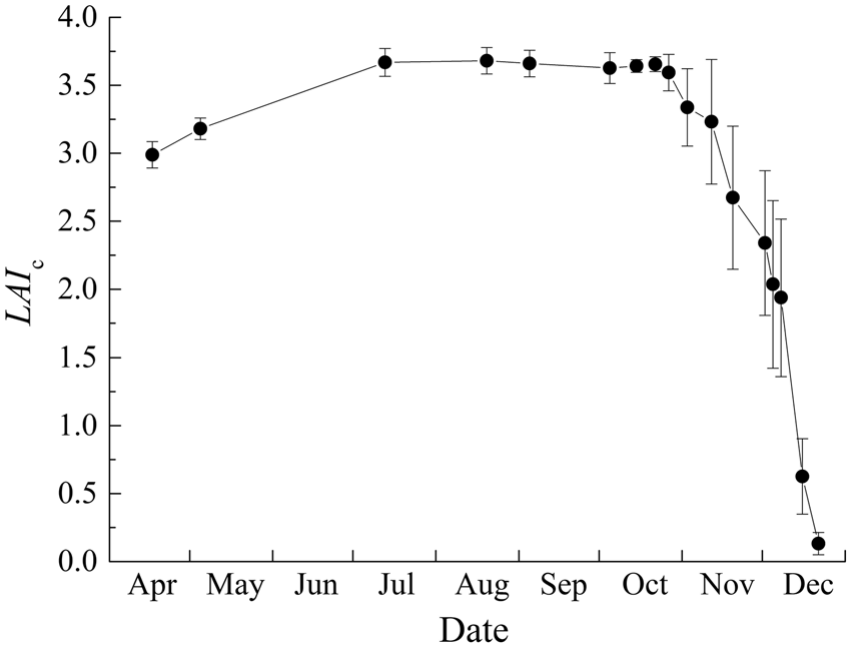
Seasonal variation of the canopy leaf area index (*LAI*_c_) of *L*. *formosana* (Data collected in 2014 and 2015 are combined. The error bar for each data point is the standard deviation from the measurements of six trees).

The leaves of *L*. *formosana* usually sprout in March, with the start of the growing season in the study area. Leaf color changes over time from light green in early spring (March–April) to deep green in summer (June–July), and finally it turns to reddish in autumn, lasting until the end of December. The leaf life span is as long as ~10 months while other deciduous trees (such as *Quercus fabri*) in the study area are completely leafless at the end of November. *L*. *formosana* leaves also sprout earlier than some other deciduous trees. A quick increase in canopy leaf area index (*LAI*_c_) occurs in spring during its early leaf development period (Fig. 1). The *LAI*_c_ maintains a relatively constant value in and after June, which means that *L*. *formosana* leaves are fully expanded around this time. The *LAI*_c_ decreases rapidly after leaves senescing and shedding in November, but before mid-December, the *LAI*_c_ value is still relatively high (*LAI*_c_ > 0.5; approximate 17% of the maximum *LAI*_c_ value in summer) (Fig. 1). This indicates that a large number of red leaves remain on *L*. *formosana* trees after about two months into senescence.

### Leaf gas exchange and photosynthetic capacity

**Figure 2:**
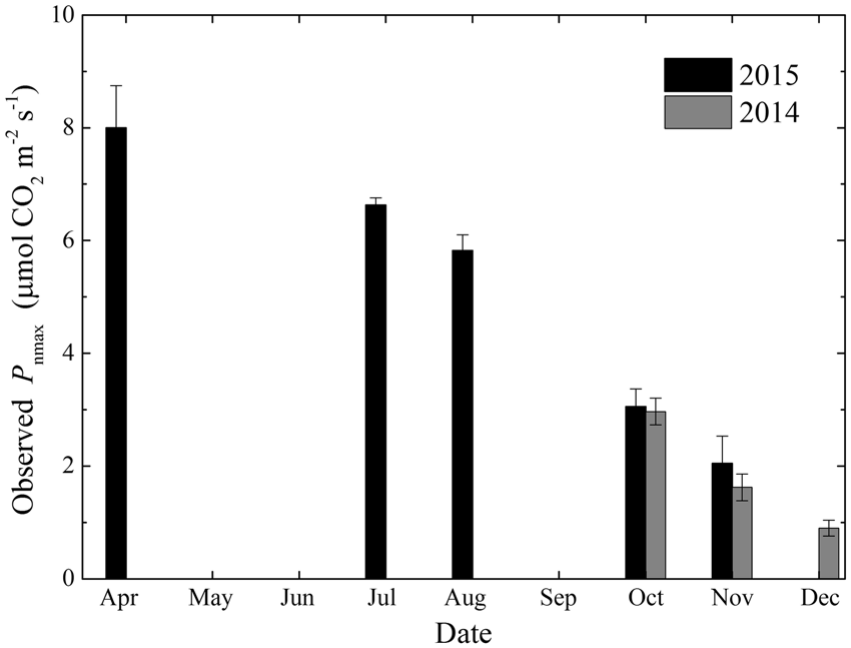
Observed monthly mean maximum rates of net photosynthesis (*P*_nmax_, means ± SE) calculated from the *P*_n_-*I* curves during the measurement period from October 2014 to November 2015.

*L*. *formosana* leaves are most sensitive to photosynthetic photo flux density (PPFD or *I*) in April when leaves are new. The observed maximum rate of net photosynthesis (*P*_nmax_) is highest in April, and then decreases slightly to a mean value of 6.2 μmol CO_2_ m^−2^ s^−1^ in July and August when leaves are mature (Fig. 2). The observed *P*_nmax_ decreases significantly (P = 0.000006) when leaves start senescing and turning red in November. But the red leaves still maintain a positive *P*_n_ even in the later stage (early December) of leaf senescence, with observed *P*_nmax_ around 0.9 μmol CO_2_ m^−2^ s^−1^. This result indicates that the senescent red leaves maintain positive CO_2_ assimilation in late autumn and early winter.

**Figure 3:**
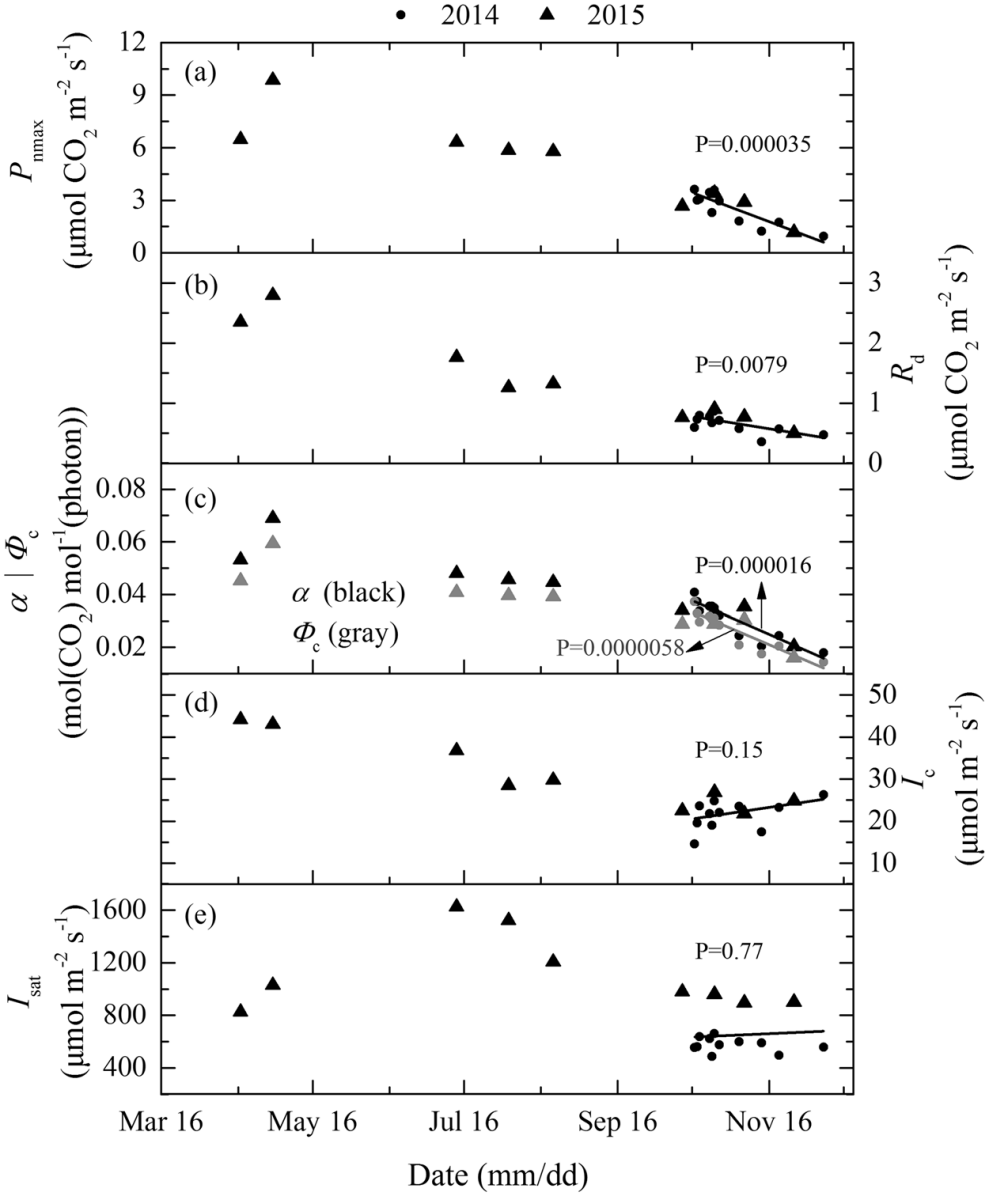
The model simulated maximum net photosynthetic rates (*P*_nmax_), dark respiration rates (*R*_d_), light compensation point (*I*_c_), light saturation point (*I*_sat_), apparent quantum yield (*α*) and the quantum yield at *I*_c_ (*Ф*_c_) of *L*. *formosana* in 2014 and 2015. Lines represent linear trend during leaf senescence, a P value smaller than 0.05 means the slope is significantly different from zero. Each data point is simulated from daily light response data composed of measurements on at least 27 leaves.

The modeled maximum rate of net photosynthesis, the dark respiration rate (*R*_d_), the apparent quantum yield (*α*), and the quantum yield at light compensation point (*Φ*_c_) decrease gradually in the leaf life span of *L*. *formosana* (Fig. 3a–c). In summer when leaves are mature, *P*_nmax_, *R*_d_, *α* and *Φ*_c_ maintain a relatively constant value (5.98 μmol CO_2_ m^−2^ s^−1^, 1.45 μmol CO_2_ m^−2^ s^−1^, 0.05 and 0.04 mol (CO_2_) mol^−1^ (photon), respectively). In autumn and winter when leaves are in senescence, *P*_nmax_, *R*_d_, *α* and *Φ*_c_ decrease significantly (Fig. 3a–c) to about 42%, 46%, 66% and 66% of that of the mature green leaves in summer, respectively. This indicates that the senescent red leaves still maintain relatively high photosynthetic capacity during this period. The model simulated light compensation point (*I*_c_) and light saturation point (*I*_sat_) remain relatively constant during leaf senescence, but both of them are lower than those of mature leaves in summer. The average *I*_c_ and *I*_sat_ of the senescent leaves are 69% and 45% of mature leaves, respectively.

**Figure 4:**
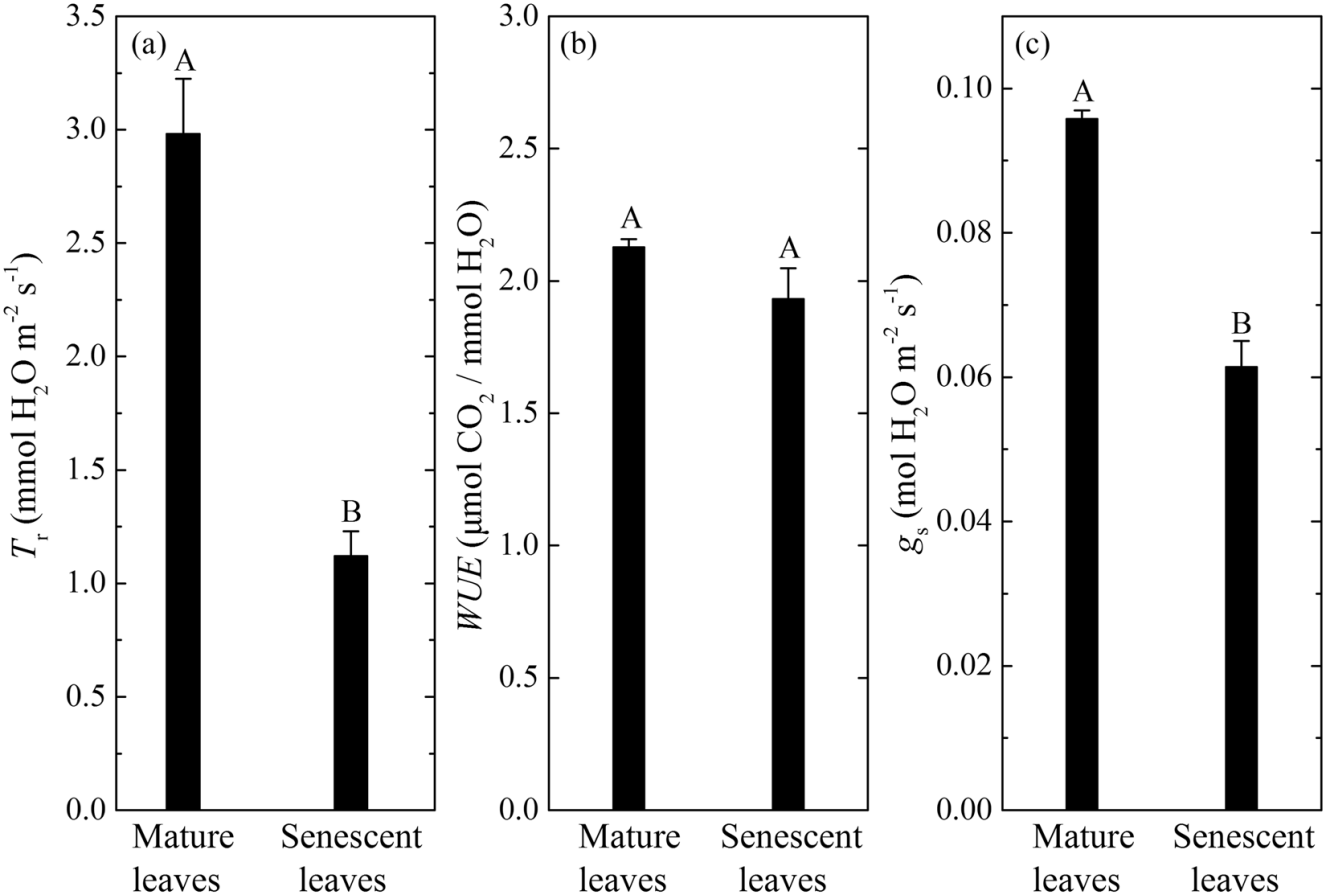
Comparison of leaf transpiration rates (*T*_r_), water use efficiency (*WUE*), and leaf stomatal conductance (*g*_s_) at light saturation point between mature green leaves (July to August) and senescent red leaves (late October to December). Values are averages ± SE (n = 3 days for mature leaves, n = 9 days for senescent leaves). Different uppercase letters on the top of the bars mean significant differences at P = 0.01 level between the mean values for mature and senescent leaves.

Leaf transpiration rates (*T*_r_), water use efficiency (*WUE*), and leaf stomatal conductance (*g*_s_) shown in Fig. 4 further demonstrate the differences in these physiological properties between senescent red leaves and mature green leaves. The *T*_r_ and *g*_s_ of green leaves show relatively high and, statistically different values (2.98 mmol H_2_O m^−2^ s^−1^ and 0.10 mol H_2_O m^−2^ s^−1^, respectively) for mature leaves (July to August) compared to those for senescent leaves (P = 0.00001 for *T*_r_, and P = 0.0003 for *g*_s_). The average *g*_s_ is about 0.061 mol H_2_O m^−2^ s^−1^ during the leaf senescence, about 64% that of mature green leaves in summer (Fig. 4c). This relatively high value of *g*_s_ indicates that the stomata of red leaves are still active (open), which contributes to maintaining a positive *P*_n_ and *T*_r_ during the leaf senescence. But the mean *WUE* of red leaves is close to that of green leaves. There is no significant difference (P = 0.365) in *WUE* between red leaves (late October to December) and green leaves (July to August).

## Discussion

**Figure 5:**
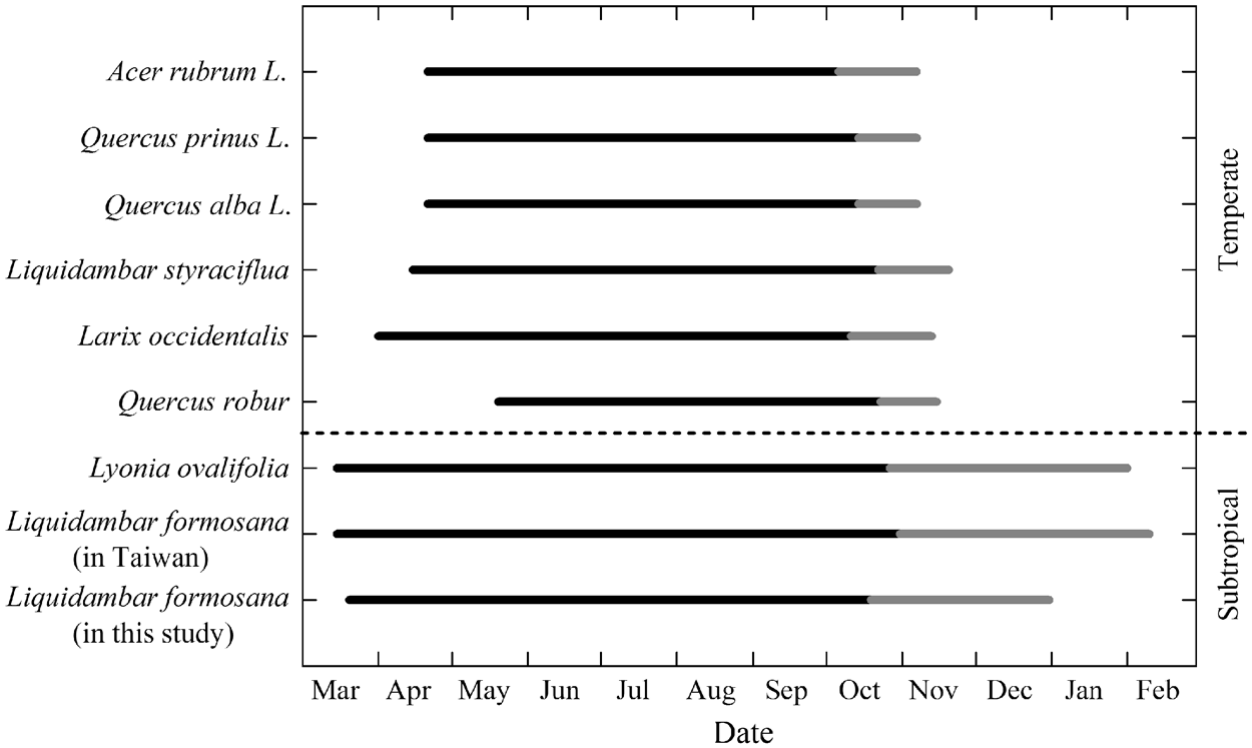
Comparison of leaf life span (estimated from leafy to leafless) between *L*. *formosana* and other subtropical and temperate deciduous tree species. Gray lines represent the leaf senescence periods for each species. Data for *Lyonia ovalifolia* are from Zhang *et al*.^9^, *Liquidambar formosana* from Wen *et al*.^42^, *Quercus robur* from Morecroft *et al*.^36^, *Larix occidentalis* from Rosenthal *et al*.^37^, *Liquidambar styraciflua* from Herrick *et al*.^16^, *Quercus alba L*., *Quercus prinus L*. and *Acer rubrum L*. from Wilson *et al*.^51^.

Leaf development and senescence is a regulated metabolic but irreversible process for plants during the course of development^15, 26–28^. Time of leaf development and senescence are influenced by environmental and internal factors^15^. Even in one forest ecosystem, different deciduous trees are different in leaf life span. These differences can have a significant impact on ecosystem process such as carbon uptake, water cycle, and annual net ecosystem production^29^. In this study, *L*. *formosana* growing season starts in March and has a long leaf span for about 10 months. This leaf span is shorter than that of the same species in Taiwan (about 11 months, growing in further south to this study area of the subtropical region of China) but longer than that of *Liquidambar styraciflua* L. (less than 8 months)^16^ in a temperate region (Fig. 5). Figure 5 shows that subtropical deciduous tree species usually have a longer leaf life span and pronounced extended leaf senescence than the temperate deciduous trees. This leaf phenological characteristic for subtropical deciduous plants is likely a result of long-term climate acclimation of these subtropical species. The prolonged growing season (compared to temperate trees) for subtropical deciduous trees means a longer time of photosynthetic activity in comparison to their temperate counterparts.

**Figure 6:**
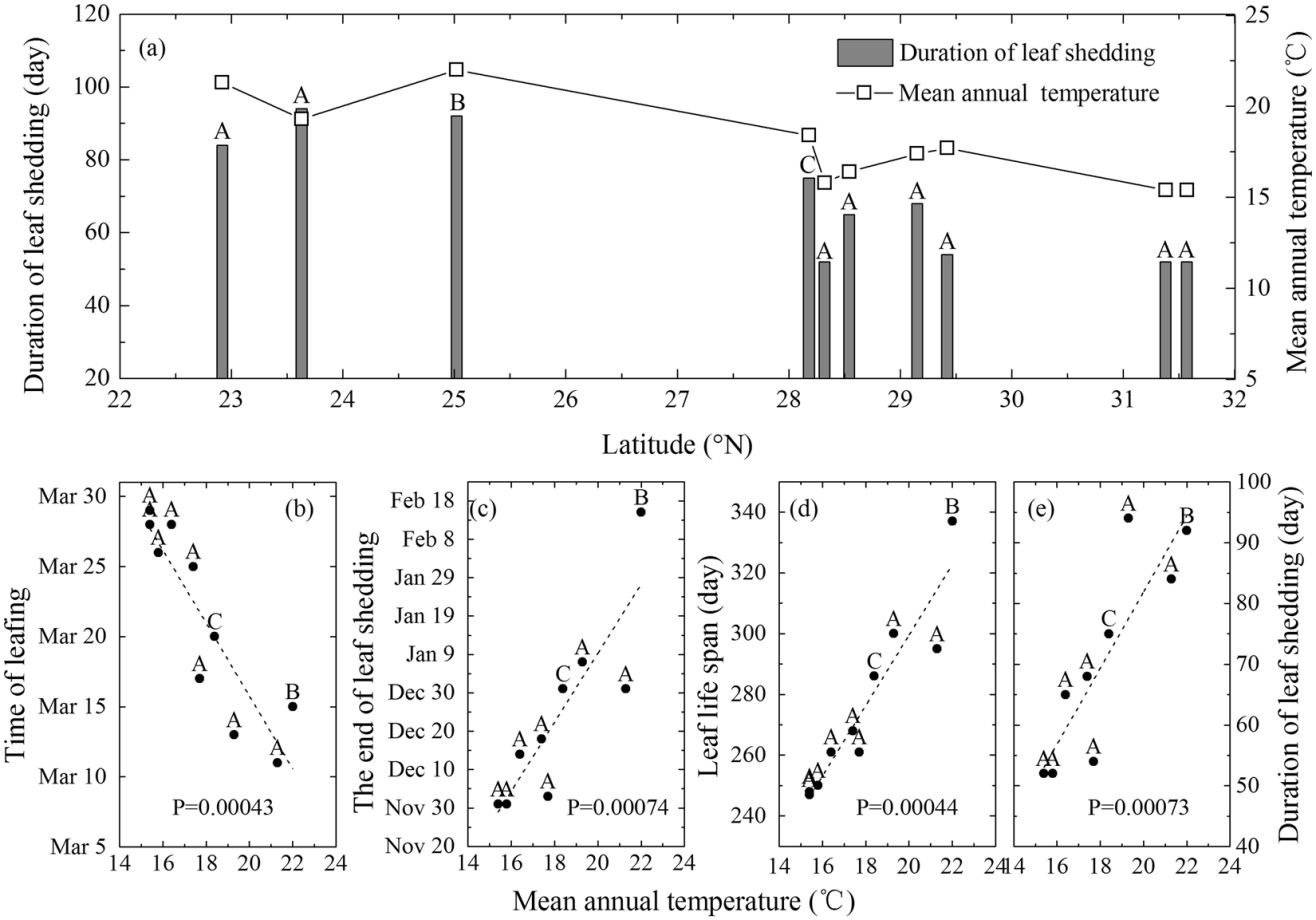
Duration of leaf shedding for *Liquidambar formosana* among different latitudes in China (**a**), and the scatter plots between time of leafing, the end of leaf shedding, leaf life span, duration of leaf shedding and mean annual temperature (**b**–**e**). Dash lines in (**b**–**e**) represent fitted linear relationships, and a P value smaller than 0.05 means the slope is significantly different from zero. The labels (A, B, and C) on the top of the bars in (**a**) and dots in (**b**–**e**) represent the data sources, A from Wang *et al*.^52^, B from Wen *et al*.^42^, and C from this study.

With a warming climate, leaf life span of deciduous species likely becomes longer (e.g., earlier onset of green-up, or extended leaf senescence, or both)^15, 18^. Yu *et al*.^18^ has reported that temperature is a main influence factor on advances of green-up and delays of leaf senescence in subtropical deciduous broadleaf forest in China. In order to find the relationship between leaf phenology of *L*. *formosana* and temperature, we correlate the duration of leaf shedding for *L*. *formosana* among different areas in China and the relationship between their phenological parameters (time of leafing, the end of leaf shedding, leaf life span and duration of leaf shedding) with mean annual temperature (Fig. 6). *L*. *formosana* trees that grow in lower latitudes show a longer duration of leaf shedding (Fig. 6a). Meanwhile, a negative correlation is showed between mean annual temperature and the time of leafing, and a positive correlation between the end date of leaf shedding and mean annual temperature (Fig. 6b,c). These results indicate that *L*. *formosana* trees living in lower latitudes, experiencing higher mean temperatures, have a longer leaf life span than those in higher latitudes. Leaf life span and duration of leaf shedding show a significant positive correlation (P = 0.0004 and 0.0007 respectively) with mean annual temperature. The difference in leaf phenology of *L*. *formosana* among different sources results from the long-term environmental acclimation, including temperature acclimation. This indicates that the leaf life span and leaf senescence (shedding) of *L*. *formosana* is sensitive to temperature, among other environmental factors (e.g., soil nutrients and water availability) and is likely to, if factored in global warming, extend in the future. In fact, autumn air temperature has increased at a rate of 0.37 °C/decade from 1970 to 2015 at the study site (Figure S1), which has possibly influenced leaf senescence of *L*. *formosana*.

Leaf senescence is a natural loss of leaf function for deciduous species and generally reflects the acclimation to the environmental conditions. Warmer ambient temperature is expected to delay autumn senescence^23^, and increases plants activity. Keenan *et al*.^30^ and Dragoni *et al*.^31^ have shown that warming-induced late autumn senescence enhances net carbon uptake in temperate evergreen and deciduous forests. But the conflicting results have been reported for boreal forest^32, 33^. In addition to temperature, other environmental variables may cause variation in leaf physiological activities. Herrick *et al*.^16^ shows that elevated CO_2_ concentration stimulates leaf photosynthesis of a temperate deciduous species (*Liquidambar styraciflua*) in late season, but does not lengthen its growing season. Leaf senescence can be accelerated or delayed in some species under drought condition^34, 35^. Overall, environmental factors are closely related to plants leaf phenology, growing season and photosynthetic function, which crucially defines how much carbon a plant assimilates during the leaf life span. Documentation of leaf phenology and photosynthetic capacity provides baseline data to investigate the possible ecosystem response to future climate change. In this study, *L*. *formosana* shows an extended leaf senescence period with positive net photosynthesis rates under the current climate condition. This evidence supports the notion of late season carbon assimilation for this subtropical deciduous species.

**Figure 7:**
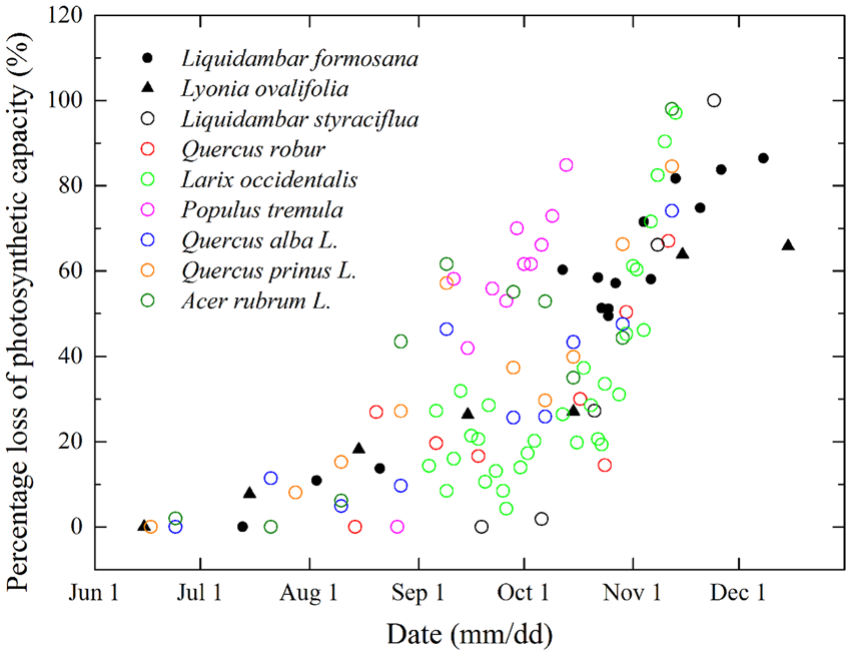
Percentage loss of photosynthetic capacity (*P*_nsat_ (light-saturated net photosynthetic rate) or *P*_nmax_) of *L*. *formosana* and other subtropical (solid symbols) and temperate (open symbols) deciduous tree species for comparison. All data are percentage loss from the maximum *P*_nsat_ in summer season. Data for *Lyonia ovalifolia* (monthly) are from Zhang *et al*.^9^, *Liquidambar styraciflua* from Herrick *et al*.^16^, *Quercus robur* from Morecroft *et al*.^36^, *Larix occidentalis* from Rosenthal *et al*.^37^, *Populus tremula* from Sun *et al*.^38^, *Quercus alba L*., *Quercus prinus L*. and *Acer rubrum L*. from Wilson *et al*.^53^.

The down-regulation in photosynthetic capacity during senescence has been documented for many deciduous tree species^9, 16, 36–38^. The results of this study show that the photosynthetic capacity (*P*_n_, *P*_nmax_, *α*, *Φ*_c_) of *L*. *formosana* senescent red leaves is lower than that of mature green leaves, but the red leaves still maintain relatively high photosynthetic capacity (about 42%, 66% and 66% of *P*_nmax_, *α* and *Φ*_c_ of the mature green leaves, respectively) during leaf senescence (Fig. 3). Figure 7 gives a further demonstration of changes in percentage loss of the photosynthetic capacity of *L*. *formosana*, in comparison to some other deciduous trees from summer to winter in the Northern Hemisphere. Of all species included in the comparison, *Lyonia ovalifolia* (Wall.) Drude and *L*. *formosana* maintain relatively high photosynthetic capacity during the senescence period, with a reduction of ca. 65% and 81%, respectively (Fig. 7).

In this study, we focus on photosynthetic capacity of *L*. *formosana* during leaf senescence. Although the net carbon gain of *L*. *formosana* cannot be accurately estimated due to a lack of night-time respiration measurements, we can conclude that *L*. *formosana* trees maintain positive daytime carbon assimilation during the extended leaf senescence. Whether or not this daytime carbon assimilation exceeds carbon loss from night-time respiration remains for future investigation. For subtropical deciduous tree species (including *L*. *formosana*), further research, in the context of global warming, is needed to investigate the responses of leaf phenology and photosynthetic capacity to climate changes.

## Methods

### Study site

**Figure 8:**
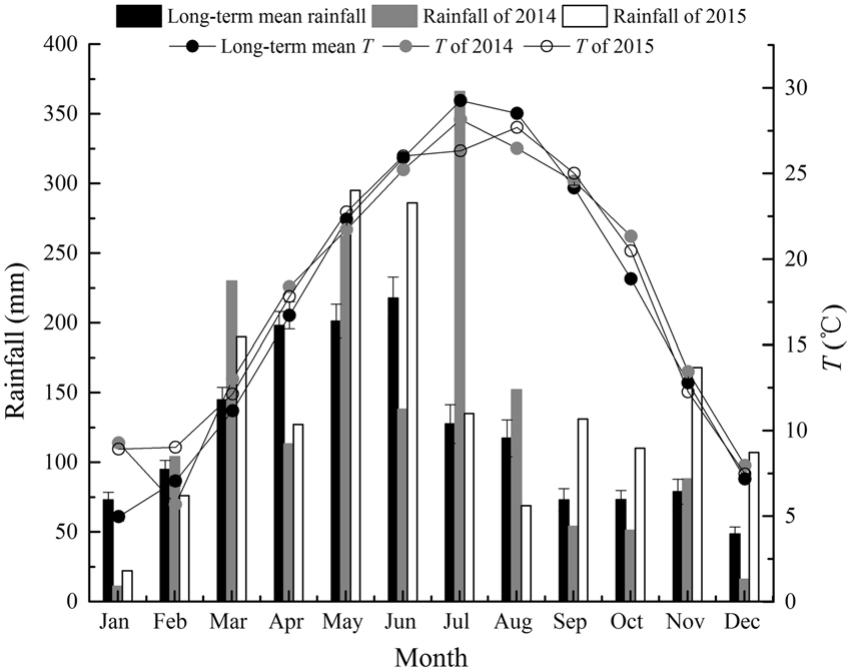
Monthly rainfall (bars) and air temperature (*T*, lines) for 2014–2015. Long-term mean monthly rainfall and *T* (means ± SE) between 1970 and 2015 are shown for comparison (Data from the Changsha weather station).

This study was performed in Yuelu Mountain, located in Changsha city in the central southern China (28°10′36″N, 112°55′58″E, 190 m above sea level). Changsha is characterized with a humid subtropical monsoon climate, with a mean annual precipitation of 1,447 ± 36 mm (mean ± SE, average of 1970–2015) and mean annual temperature of 17.4 ± 0.1 °C (mean ± SE, average of 1970–2015). Resulting from northerly cold air mass influences in the winter monsoon season, it usually has dry and cold winter. Rainfall mainly occurs in spring to early summer. Figure 8 shows the climate conditions in 2014 and 2015. Both years were wetter under the context of El Niño phenomenon. Especially for 2015, it rained more than normal from September to December. Mean monthly air temperature of 2014–2015 was lower in the summer than that of long-term mean monthly temperature and was higher in autumn and early winter than the long-term average. The soils of this site are yellowish red soils. The surface soil layer contains organic matters^39^. Forest in Yuelu Mountain is dominated by typical subtropical evergreen broadleaf trees, mixed with some deciduous trees.

*Liquidambar formosana* Hance, investigated in this study, is the dominant deciduous tree species interspersed naturally among the dominant evergreen species. This species widely distributes in subtropical deciduous broadleaf forest, and evergreen and deciduous broadleaf mixed forest^40, 41^. The leaves of *L*. *formosana* usually sprout in spring (March) and begin to turn yellow and/or red in autumn. Senescent leaves of this tree species usually last for about two months in this region. Nevertheless, in northern Taiwan (southern China), *L*. *formosana* shows a longer senescence period (more than three months)^42^.

### Leaf gas exchange

Leaf gas exchange measurements were performed on six *L*. *formosana* trees from October to December 2014, and April to November 2015. Measurements of 3–5 sun-exposed leaves for each individual tree were taken for light response curves between 0900 and 1130 hour local time on typical sunny days, using a LI-6400XT portable photosynthesis system (LI-COR, NE, USA). The photosynthetically active radiation (PAR) was provided by a red/blue light source (Li-6400-02B) connected to the system with a specific gradient of *I* (PPFD) ranging from 0 to 2000 μmol m^−2^ s^−1^ (0 to 1000 μmol m^−2^ s^−1^ for measurements from October to December 2014). At each light intensity level, we kept a minimum wait time of 120 s, and a maximum wait time of 200 s before capturing the value. For dark respiration, we kept a wait time of 200 s for a dark adaptation of leaves prior to capturing its value. The air flow rate was set to 500 μmol s^−1^, chamber temperature was kept constant at 25 ± 1 °C and chamber CO_2_ concentration was kept at 400 ± 2 μmol CO_2_ mol^−1^.

Responses of net photosynthesis rates (*P*_n_) to *I* were fitted with a recently published photosynthetic light response model modified from the rectangular hyperbolic model^43^, as shown in

1 $${P}_{n}=\alpha \frac{1-\beta I}{1+\gamma I}I-{R}_{{\rm{d}}}$$

where *α* is apparent quantum yield, *R*_d_ is dark respiration rate, *β* and *γ* are coefficients which are independent of *I*.

Then, the maximum rate of net photosynthesis (*P*_nmax_), light compensation point (*I*_c_), and light saturation point (*I*_sat_) can be calculated from the model. Moreover, this new model also calculates the quantum yield at *I*_c_ (*Φ*_c_)^43, 44^ by

2 $${{\Phi }}_{{\rm{c}}}={\rm{\alpha }}\frac{1+(\gamma -\beta ){I}_{{\rm{c}}}-\beta \gamma {I}_{{\rm{c}}}^{2}}{{(1+\gamma {I}_{{\rm{c}}})}^{2}}$$

Measurements in a day were averaged for each light intensity level, and then were fitted with equation (1). The parameters calculated from the light response model provide quantitative representation of the photosynthesis capacity and efficiency^45, 46^.

Measurements of leaf gas exchange also include water vapour, which can be used to calculate leaf transpiration rates. Leaf-scale water use efficiency (*WUE*) is defined as the ratio between leaf net photosynthesis rate (*P*_n_) and transpiration rate (*T*_r_).

### Leaf area index for the canopy of an individual tree

Leaf area index (*LAI*) is a key parameter reflecting the structure of plant canopy. Unfortunately, *LAI* is difficult to quantify accurately although many methods have been proposed^47–49^. For the purpose of this study, leaf area index for the canopy^50^ of a single tree (*LAI*_c_) is used to reflect canopy characteristic and number of leaves retained on the studied *L*. *formosana* trees. We adopted a regular camera-based method developed by Pekin and Macfarlane^49^. The method estimates crown cover and *LAI*_c_ based on the digital photography. *LAI*_c_ is calculated by the following equations:

3 $$\omega =1-{f}_{{\rm{f}}}/{f}_{{\rm{c}}}$$

4 $$LA{I}_{{\rm{c}}}=-{f}_{{\rm{c}}}\,\mathrm{ln}({\rm{\omega }})/k$$

where *ω* is the porosity of an image, *f*_f_ and *f*_c_ are the foliage cover and crown cover of an image, respectively. Parameter *k* is a zenithal light extinction coefficient.

Digital photos were taken in eight directions under the tree crown weekly during the measurement period. The photo time avoided direct sunshine from the top and strong windy conditions. The camera base was kept perpendicular to the tree trunk when a photo was taken. All images data were analyzed by a program written in MATLAB, kindly provided by Prof. Craig MacFarlane from The University of Western Australia. The *LAI*_c_ of each individual tree was calculated from the average of its eight directions.

### Statistical analysis

Statistical analyses were conducted with SPSS 13.0 software package (SPSS Inc., USA) to examine the significance of difference between data. For example, the difference in mean *P*_nmax_, *α*, *R*_d_, *Φ*_c_, *I*_c_ and *I*_sat_ between mature green leaves and senescent red leaves were tested by one-way ANOVA. Significance was found when P < 0.05.

## Electronic supplementary material

Supplementary Figure
