# Supplementary material for: Photosynthetic capacity of senescent leaves for a subtropical broadleaf deciduous tree species Liquidambar formosana Hance
Source: Sci Rep. 2017 Jul 24;7:6323. doi: 10.1038/s41598-017-06629-7 (PMC5524682; doi:10.1038/s41598-017-06629-7)

Photosynthetic capacity of senescent leaves for a subtropical broadleaf deciduous tree species *Liquidambar formosana* Hance

Zidong Luo[1], Huade Guan[2], Xinping Zhang[1*], Na Liu[1]

[1]College of Resource and Environment Science, Hunan Normal University, Changsha 410081, China

[2]School of the Environment & National Centre for Groundwater Research and Training, Flinders University, Adelaide, SA 5001, AUS

[*]Corresponding author: Dr. Xinping Zhang, College of Resources and Environmental Sciences, Hunan Normal University, 410081 Changsha, Hunan, China.

E-mail: zxp@hunnu.edu.cn

Supplementary Figure

Figure S1 Variation of air temperature in September (a), October (b), November (c) and autumn (d) from 1970 to 2015. A P value smaller than 0.05 means the slope is significantly different from zero.

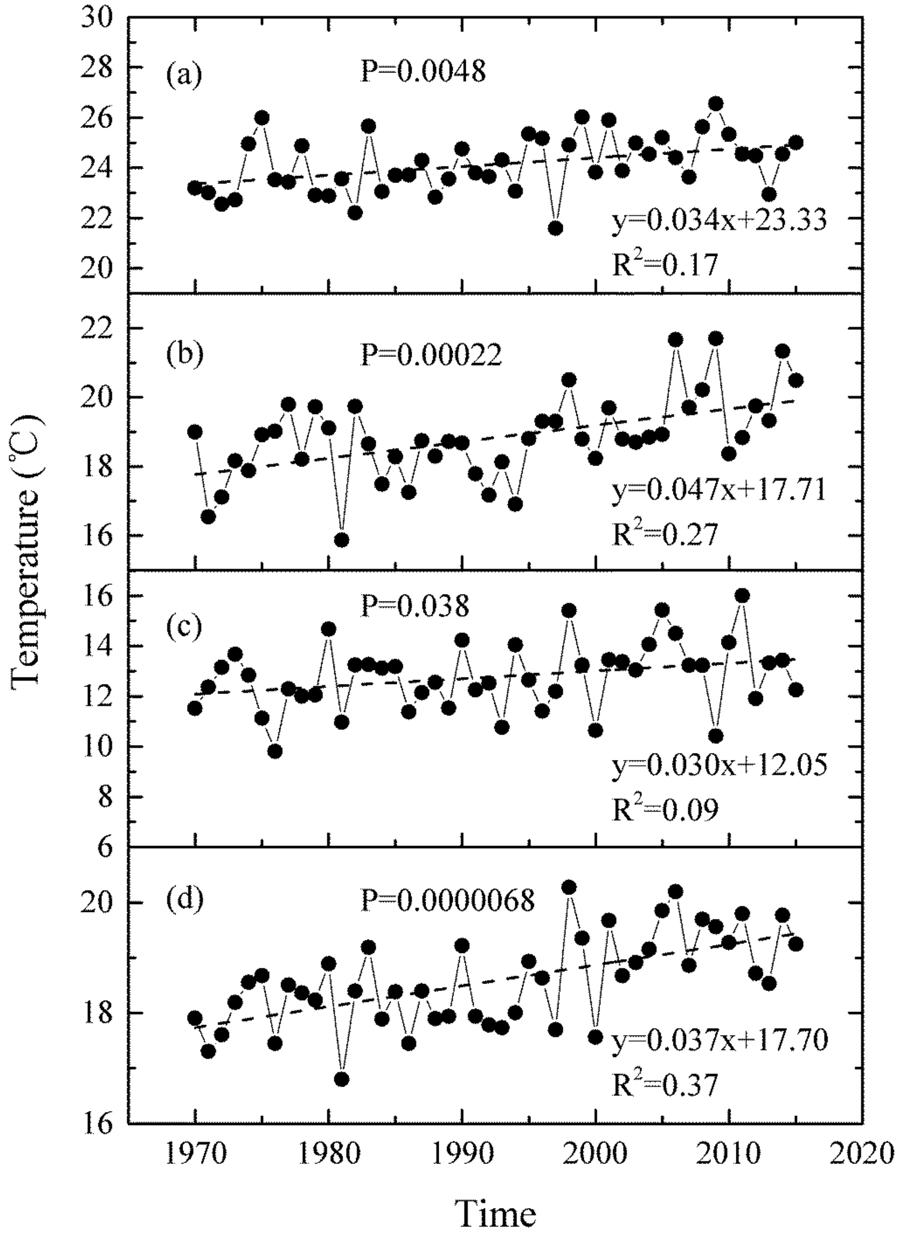

Supplement: Supplementary file 1 — Supplementary Figure [file 41598_2017_6629_MOESM1_ESM.pdf]
